# Proceedings of the International Cancer Imaging Society Meeting and 21st Annual Teaching Course: Boston, MA, USA. 12-14 September 2022

**DOI:** 10.1186/s40644-022-00479-x

**Published:** 2022-09-09

## Oral presentations

### O1. Impact of image resampling voxel size on the reproducibility of radiomic features in brain metastases

#### Yixin Wang^1,2,3^, Zongtao Hu^1,3^, Xiuli Xu^3^, Yongkang Zhang^3^, Hongzhi Wang^1,2,3^

##### ^1^Anhui Province Key Laboratory of Medical Physics and Technology, Institute of Health and Medical Technology, Hefei Institutes of Physical Science, Chinese Academy of Sciences, Hefei, 230031, P. R. China, ^2^University of Science and Technology of China, Hefei 230026, China/P. R. China, ^3^Department of Oncology, Hefei Cancer Hospital; Chinese Academy of Sciences, Hefei 230031, China/P. R. China

###### **Correspondence:** Hongzhi Wang (wangyx0823@163.com)

Aim

The reproducibility of radiomic features could depend on image resampling voxel size. However, the impact of different resampling voxel sizes on multi-parameter magnetic resonance imaging (MRI) radiomic features in patients with brain metastases remains unknown. In this study, we aimed to investigate the reproducibility of radiomic features against different resampling voxel sizes among different MRI sequences, different regions of interest (ROIs), and different feature classes and image processing types.

Materials and Methods

Images of multi-parameter MRI in patients with brain metastases were collected. Four different voxel sizes were used to resample multi-parameter MRI. In each resampled or original MRI sequence, 1015 resampling or original radiomic features were extracted from the tumour or oedema area. Intraclass correlation coefficient (ICC) was used to assess the reproducibility of resampling radiomic features compared with original radiomic features. Features with ICC greater than or equal to 0.75 were defined as robust features.

Results:

The number of robust features was lower when the resampling voxel size became bigger. The tumour contained more robust radiomic features than the oedema area. Radiomic features of diffusion-weighted images (DWI) were more robust than features of other MRI sequences. Among all MRI sequences, radiomic features of shape feature class and original image type were the most robust.

Conclusions

The resampling voxel size impacted the reproducibility of multi-parameter MRI-based radiomic features in patients with brain metastases. The summary of the robust radiomic features against resampling voxel size may be helpful for radiomic studies of brain metastases.

### O2. CT/MR texture analysis predictive value for tumour aggressiveness of liver metastases from neuroendocrine tumours

#### S. Picchia, M.A. Bali, M. Rengo, P. Flamen, A. Deleporte, I. Karfis, A. Hendlisz, A. Acquisto, I. Carbone, A. Laghi

##### Hopital Jules Bordet, Rue Meylemeersch 90, 1070 Bruxelles, Belgium

###### **Correspondence:** S. Picchia (simona.picchia87@gmail.com)

Aim

The study's aim is to evaluate the predictive value of texture analysis (TA) metrics calculated for liver metastases from Neuroendocrine Tumours (NET) on CT and MR in discriminating tumour aggressiveness based on PET-evaluation and histological tumour grade.

Materials and methods

Patients with NET liver metastases previously investigated with FDG and Octreo PET were included in this multicentric retrospective study. TA metrics were calculated by drawing the whole lesion volume ROI on two liver metastases on contrast-enhanced CT (CE-CT) or ADC maps using a semi-automated tool (3D slicer). Inclusion criteria were: 1) histologically proven NET; 2) liver metastases of >/= 2cm detected on CE-CT or DWI-MR, Octreo and FDG PET before treatment. Patients were divided into two groups according to tumour aggressiveness based on PET (high aggressiveness = positivity on FDG PET, low aggressiveness = positivity on Octreo PET and negativity on FDG PET) and on histological grade (high aggressiveness = G3, low aggressiveness = G1-G2). A logistic regression model (software Weka) was used to assess the association between TA and tumour aggressiveness.

Results

Final study population consisted of 50 patients (M/W= 30/20; mean age=48). The CT-TA discriminated less aggressive from more aggressive PET-CT based lesions with an AUC=0.8 and 0.6 and histological grade-based with an AUC=0.9 and 0.5 respectively. The MR-TA discriminated less aggressive from more aggressive PET based lesions with AUC=0.8 and 0.5 and histological grade based with an AUC=0.8 and 0.5 respectively.

Conclusions

CT and MR TA showed good accuracy to identify less aggressive compared to more aggressive lesions based on PET and histological characteristics respectively.

### 03. Renal cell carcinoma with sarcomatoid differentiation: MRI features and their association with survival

#### M. Cheng^1,2^, C. Duzgol^1^, T.H. Kim^1^, S. Ghafoor^3^, A.S. Becker^1^, P.I. Causa Andrieu^1^, H. Jiang^1^, A.A. Hakimi^1^, H.A. Vargas^1^, S. Woo^1^

##### ^1^Memorial Sloan Kettering Cancer Center, New York, NY, USA, ^2^Massachusetts General Hospital, Boston, MA, USA, ^3^University Hospital Zurich, Zurich, Switzerland

###### **Correspondence:** S. Woo (woos@mskcc.org)

Aim

To evaluate MRI features of sarcomatoid renal cell carcinoma (RCC) and their association with survival.

Materials and Methods

This retrospective single-centre study included patients with sarcomatoid RCC who underwent MRI before nephrectomy during 2003─2019. On MRI, three radiologists reviewed tumour size, non-enhancing areas, adenopathy and volume/percentage of T2 low signal intensity areas (T2LIA). Relevant clinicopathological factors were documented. Kaplan-Meier method and univariate/multivariate Cox proportional-hazards regression model was used for survival analysis.

Results

59 patients (median age 62 years) were included. T2LIAs were present in 43 (72.9%) patients. At univariate analysis, clinicopathological factors associated with shorter survival were: size >10 cm (hazard ratio [HR] 2.44, 95%CI 1.15-5.21; p=0.02), metastatic lymph nodes (HR 2.10, 95%CI 1.01-4.37; p=0.04), non-focal extent of sarcomatoid differentiation (HR 3.30, 95%CI 1.55-7.01; p<0.01), subtypes other than clear cell/papillary/chromophobe (HR 3.25, 95%CI 1.28-8.20; p=0.01), and baseline metastasis (HR 5.04, 95%CI 2.40-10.59; p<0.01). Age, gender, ethnicity, stage and papillary/chromophobe subtypes were not associated with survival (p= 0.21-0.92). MRI features associated with shorter survival were: adenopathy (HR 2.24, 95%CI 1.16-4.71; p=0.01) and volume of T2LIA (>3.2 mL, HR 4.22, 95%CI 1.92-9.29; p<0.01). Non-enhancing areas, size, and percentage of T2LIA were not associated with survival (p=0.05-0.37). At multivariate analysis, baseline metastasis (HR 6.89, 95%CI 2.79-16.97; p<0.01), subtypes other than clear cell/papillary/chromophobe (HR 9.50, 95%CI 2.81-32.13; p <0.01) and greater volume of T2LIA (HR 2.51, 95% CI1.04-6.05; p=0.04) remained independently associated with survival.

Conclusions

T2LIAs were present in approximately two-thirds of sarcomatoid RCCs. Volume of T2LIA along with clinicopathological factors were associated with survival.

### O4. Inter-reader and inter-criteria agreement of immune-specific response criteria (irRECIST & iRECIST)

#### K. Ruchalski^1^, H.J. Kim^1^, R. Dewan^1^, M. Douek^1^, V. Sai^1^, B. Villegas^1^, K.P. Wong^1^, A. Lisberg^2^, J Goldman^2^, J. Goldin^1^, E. Garon^2^, D. Aberle^1^

##### ^1^David Geffen School of Medicine at UCLA; Department of Radiological Sciences, ^2^David Geffen School of Medicine at UCLA; Division of Hematology and Oncology, 10833 Le Conte Ave, Los Angeles, CA 90095, USA

###### **Correspondence:** K. Ruchalski (kruchalski@mednet.ucla.edu)

Aim

RECIST 1.1 can underestimate treatment benefits of immunotherapy, with irRECIST and iRECIST accounting for atypical responses. Our objective is to compare inter-reader agreement for time to progression (TTP) between RECIST 1.1, irRECIST, and iRECIST and inter-criteria agreement in progressive disease (PD).

Methods

A retrospective study of advanced NSCLC patients treated with pembrolizumab was conducted at our institution as part of the KEYNOTE-001 study. All trial imaging was interpreted by two radiologists. RECIST 1.1, irRECIST, and iRECIST categorical responses and agreement for PD were compared by proportion of agreement.

Results

Of 98 patients, 77 had baseline and subsequent imaging for 5.8 mean timepoints with 42.9 weeks of follow-up. Of these, 44 patients had imaging after iUPD for confirmation and were analysed. Readers agreed on TTP in 29/44 (65.9%) RECIST, 25/44 (56.8%) irRECIST, 31/44 (70.5%) iRECIST. Proportion of agreement (95% confidence intervals) was not significantly different; RECIST (61.8-90.2), irRECIST (55.6-87.1), iRECIST (68.0-93.8). irRECIST and iRECIST concordance for PD occurred in 81.8%, 79.5% cases for reader 1, 2. Discordance in PD occurred in 8, 9 cases for reader 1, 2. irRECIST PD with only iUnconfirmed PD by iRECIST occurred in 6, 7 cases for reader 1, 2. iConfirmed PD by iRECIST without PD by irRECIST as a result of non-target progression occurred in 2, 2 cases for reader 1, 2.

Conclusions

Reader agreement was not significantly different when applying RECIST, irRECIST or iRECIST. Inter-criteria disagreement in PD can occur due to unconfirmed PD by iRECIST or non-target progression not captured by irRECIST.

### O5. Semantic integration and harmonisation of clinical data in The Cancer Imaging Archive

#### J.P. Bona, J. Utecht, M. Brochhausen, Fred W. Prior

##### Department of Biomedical Informatics, University of Arkansas for Medical Sciences, Little Rock Arkansas, USA

###### **Correspondence:** J.P. Bona (jpbona@uams.edu)

Aim

The Cancer Imaging Archive (TCIA) de-identifies and hosts a large publicly available archive of images of cancer. The Platform for Imaging in Precision Medicine (PRISM) initiative provides a modular open-source technology platform for imaging-based precision medicine. A key component of PRISM is its novel approach to semantically harmonising and integrating clinical data accompanying imaging data collections. This abstract describes the semantic integration of these data and user-facing software that uses these integrated data.

Methods

Many TCIA image collections include supplementary clinical data, providing valuable information about the subjects. PRISM semantically integrates these data using representations built on open biomedical ontologies. We first catalogued the clinical data, creating the PRISM data catalogue. We constructed ontology-based knowledge patterns to represent key elements. We used these to transform and load the data into a triple store database. We built an API that generates SPARL queries over this knowledge graph. Finally, we built the PRISM semantic cohort builder tool.

Results

We published semantically harmonised TCIA clinical data as an ontology-aligned knowledge graph. We have also built and deployed a REST API and the semantic cohort builder, a custom-built software tool allowing non-technical users to search, explore, and aggregate information about subjects in imaging collections using the semantically transformed clinical data.

Conclusion

We have made clinical data from public imaging data collections in TCIA findable, accessible, interoperable, and reusable (FAIR). The user-friendly semantic cohort builder tool facilitates search, exploration, and use of those data.

### O6. Diagnostic significance of Ga 68 FAPI PET/CT in hepatobiliary malignancies

#### S.S. Shivalingappa, K.G. Kallur, A.G. Nadezhda Niyarah, A.K. Mahesh, R. Avinash Kesari, G.R. Prashanth, S.S. Rajkumar, A.P. Praveen

##### HealthCare Global Enterprises. Bengaluru, Karnataka, India

###### **Correspondence:** S.S. Shivalingappa (drshiv.radiologist@gmail.com)

Aim

1. To assess the diagnostic value of 68Ga-FAPI-04 PET/CT for the detection and characterization of primary hepatic malignancies
2. To correlate the SUV max, TLG and MTV with tumour size and stage of disease.

Materials and Methods

Desmoplastic, stroma-rich hepatobiliary tumours express an increased number of cancer associated fibroblasts which are proved necessary for metastasis and aggressive behaviour of these tumours. So, accurate quantification (SUV max, TLG and MTV) on 68Ga-FAPI-04 scan will signify malignant nature of the tumour. The study included a total of 55 patients with suspicious hepatobiliary lesions which included different grades of hepatocellular carcinoma and different types of cholangiocarcinoma. Lesion characterisation was determined by post-surgical histology or biopsy. None of the patients had received any anti-tumour treatment prior to the PET/CT scans.

Results

The average SUV max was seen to be 6.45+/-1.2 with no significant difference between the individual tumour subgroups. The SUV max value, TLG and MTV were proportional to the size of tumour and tumour grade.

Conclusion

The study demonstrates the high diagnostic accuracy of 68 Ga-FAPI PET in the diagnosis of primary hepatobiliary malignancies. The proportional rise in SUV value with respect to tumour size also allows for pre-surgical staging.

Ethics Committee approval

Study was started after obtaining approval from HCG Institutional Ethics Committee.

### O7. Stereotactic body radiotherapy (SBRT) for primary and secondary liver tumours: Efficacy and evaluation of response

#### F. Castagnoli^1^, A. Villanacci^2^, M. Bertuletti^2^, A. Castaldo^2, 3^, D.M. Koh^1^, L. Grazioli^2^

##### ^1^Royal Marsden Hospital, Sutton, UK, ^2^ASST Spedali Civili, Brescia, IT, ^3^“Federico II” University, Naples, IT

###### **Correspondence:** F. Castagnoli (francy-cast@hotmail.it)

Aim

To evaluate early changes in ADC values and size of HCC and liver metastases treated with SBRT.

Materials and Methods

29 patients with HCC (n=18) and metastatic lesions (n=11), treated with SBRT, who underwent baseline gadoxetate-enhanced MRI within 4 weeks before treatment and follow-up MRI within 3 months after finishing SBRT were prospectively enrolled (October 2014-January 2022). Two radiologists evaluated, in consensus, tumour size (∂) and ADC values of all lesions. Local control (LC) was evaluated after a median follow-up of 2.1 years. Delta (Δ)∂ and ΔADC were correlated using Spearman correlation coefficient. Δ∂ and ΔADC were compared (Mann-Whitney test) between patients with (n=26) and without (n=3) LC and between HCC and metastases.

Results

The median HCC ADC (10^-3^mm^2^/s) and dimension were 0.963 and 25.6 mm for baseline; 1.244 and 19.8 mm for follow-up. For metastases, the median ADC (10^-3^mm^2^/s) and dimension were 0.990 and 20.7 mm for baseline; 1.427 and 18.7 mm for follow-up. The ADC values increased by 29.1% for HCC and 44.2% for metastases (p=0.04), whereas tumour size decreased by 22.6% and 9.6%, respectively (p=0.01). There was no correlation between the increase in ADC values and decrease in lesion dimension in both groups (p=0.41 and p=0.2). There was no significant difference in ΔADC and Δ∂ between patients with and without LC (p=0.7, p=0.2) and between HCC and metastases (p=0.6, p=0.1).

Conclusion

ADC values showed greater increase while tumour diameters showed smaller decrease in metastases compared with HCC. SBRT showed excellent LC for both HCC and metastases, which were not associated with the degree of early ADC and diameter change after treatment.

### O8. Fat-containing adnexal masses: Solid tissue size and fat distribution for refining O-RADS MRI scoring system

#### M. Cheng^1,2^, P.I. Causa Andrieu^1^, T.H. Kim^1^, N. Gangai^1^, Y. Sonoda^1^, H. Hricak^1^, Y. Lakhman^1^, H.A. Vargas^1^, S. Woo^1^

##### ^1^Memorial Sloan Kettering Cancer Center, New York, NY, USA, ^2^Massachusetts General Hospital, Boston, MA, USA

###### **Correspondence:** S. Woo (woos@mskcc.org)

Aim

To evaluate O-RADS MRI scoring system for fat-containing adnexal masses by investigating methods for quantifying volume of solid tissue and associations between fat distribution and malignancy.

Materials and Methods

This retrospective single-centre study included patients with fat-containing adnexal masses on MRI during 2008-2021. Two radiologists independently reviewed overall size (Size_overall_), size of any solid tissue (Size_anysolid_), size of solid tissue that was not a Rokitansky nodule (Size_non-Rokitansky_), and fat distribution. Reference standard was pathology or follow-up at >6 months. Wilcoxon test, Fisher-exact test, and ROC analysis were performed.

Results

205 women (median age 35 years) with 180 benign and 25 malignant lesions were included. Size_overall_ (R1, 9.9 cm vs 5.6 cm; R2, 12.4 cm vs 5.7 cm), Size_anysolid_ (R1, 5.1 cm vs 1.2 cm; R2, 3.2 cm vs 0.0 cm), Size_non-Rokitansky_ (R1, 5.1 cm vs 0.0 cm; R2, 3.1 cm vs 0.0 cm), and fat distribution were significantly different between malignant and benign lesions (p<0.01). Area under ROC was greatest using Size_non-Rokitansky_ (R1, 0.84; R2, 0.86) compared to Size_overall_ (R1, 0.80; R2, 0.83) and Size_anysolid_ (R1, 0.80; R2, 0.81) without statistical significance (p=0.49–0.98). Optimal cut-offs using Size_non-Rokitansky_ were ≥1.2 cm (R1) and ≥1.0 cm (R2), yielding sensitivities and specificities of 0.72 and 0.93 for R1 and 0.76 and 0.95 for R2. Scattered fat pattern was present in 90% of immature teratomas.

Conclusions

In fat-containing adnexal masses, overall size, size of (any or non-Rokitansky nodule) solid tissue, and fat distribution were significantly different between benign and malignant lesions.

### O9. Assessment of early response to neoadjuvant chemotherapy in multi-site high-grade serous ovarian cancer using hyperpolarized-13C-MRI

#### V. Bura^1,2,3^, L. Beer^1,4^, M.A. McLean^1,2^, A. Frary^1^, M. Locke^1^, C. Brodie^2^, M. Jimenez-Linan^1,2^, F.A. Gallagher^1,2^, K Brindle^2^, R Woitek^1,2,4^, E Sala^1,2^

##### ^1^University of Cambridge, Cambridge, UK, ^2^Cancer Research UK Cambridge Institute, Cambridge, UK, ^3^County Clinical Emergency Hospital, Cluj-Napoca, Romania, ^4^Medical University, Vienna, Austria

###### **Correspondence:** V. Bura (vb423@cam.ac.uk, vlad.t.bura@gmail.com)

Aim

Hyperpolarized-[1-13C]pyruvate-MRI (HP13C-MRI) is an emerging imaging technique to detect in-vivo tumour lactate production. The major clinical challenge in advanced high-grade serous ovarian cancer (HGSOC) is to identify non-responders to neoadjuvant-chemotherapy (NACT) early in the management course, in order to guide therapy. This exploratory study is the first to investigate the potential of HP13C-MRI for assessing early response to NACT in HGSOC patients.

Materials and Methods

Patients with biopsy-proven HGSOC, scheduled for NACT, underwent 3T-HP13C-MRI, before and after the administration of first cycle of NACT (NACT-C1). Tumour-derived [1-13C]pyruvate-to-[1-13C]lactate conversion rate (kPL) and %kPL-change (from before to after NACT) was calculated. Tumour histopathological response to NACT was assessed on surgical sections by an expert gynaecological pathologist. Consecutive sections were used for immunohistochemical analysis of monocarboxylate transporters (MCTs, influx/efflux lactate-transporters) 1&4 membrane-expression and for LDHA quantification using liquid chromatography mass-spectrometry analysis.

Results

The study included 8 patients with 14 lesions. Decreased lactate labelling was detected after NACT-C1 in 9/14 lesions with pathological response, while no decrease was observed in 5/14 lesions with no response after NACT. kPL correlated positively with LDHA (rs=0.60,P=0.02) and negatively with tumour-stroma MCT4(+) ratio (rs=0.70,P=0.004). kPL and %kPL-change were significantly different for responder versus non-responder lesions (P<0.01).

Conclusions

In our study, decreased lactate labelling was associated with pathological response, while no significant decrease was shown in non-responder lesions, supported by relevant molecular data. To assess early response to NACT in patients with HGSOC, metabolic signatures derived from HP13C-MRI measurements could be used as a non-invasive stratification tool to differentiate responders from non-responders.

## Poster abstracts

### P1. Imaging in thoracoabdominal extraosseous sarcomas with histopathological and immunohistochemistry correlation

#### N. Alemao, S. Swamy, A. Kesari, M. Kumar, S. Sampangi, K. Kallur

##### HCG Hospital, Bangalore, India

###### **Correspondence:** N. Alemao (niyarahalemao99@ymail.com)

Learning Objectives

Sarcomas, derived from mesenchymal tissues constitute around 1-2% of cancers amongst adults. The challenging diagnosis of sarcomas present opportunities for evaluation and validation of new imaging techniques. Abdominal sarcomas particularly have poor prognosis, mainly due to the non-specific presentation and late diagnosis. Imaging can aid in early diagnosis and improve the 5-year survival rate. In this case series, we aim to study the PET/CT (positron emission tomography/computed tomography) and MRI (magnetic resonance imaging) features of abdominal and thoracic sarcomas and to correlate the imaging findings with histopathological and immunohistochemistry features.

Content Organisation

A total of 12 cases of thoracoabdominal sarcomas who underwent PET/CT and MRI along with histopathological and immunohistochemistry evaluation were included in the study. These included sarcomas involving the mesentery, abdominal hollow viscera, liver, mediastinum and retroperitoneal areas. These were endometrial stromal sarcoma, pericardial angiosarcoma, suprarenal leiomyosarcoma, retroperitoneal sarcoma, malignant fibrous histiocytoma, rhabdomyosarcoma, epithelioid angiosarcoma, retroperitoneal and mediastinal Ewing’s sarcoma and retroperitoneal liposarcoma. PET/CT, though non-specific, identified most of the abdominal sarcomas in the study with a size larger than 2 cm. For smaller lesions, MRI proved to be a better modality to aid in identification.

Conclusion

Carcinomas metastasise through the lymphatic route and the haematogenous route. While mesenchymal sarcomas frequently enter the bloodstream directly causing lung or liver metastases, but rarely result in lymph node metastases. Definitive diagnosis in these cases was confirmed by histopathology but appearances on CT and MRI may reflect some features of sarcomas. Hence familiarity with radiological finding of abdominal visceral sarcomas might aid in the diagnosis.

### P2. Imaging: Support enabling quality of life improvements for people facing end of life

#### J. Eastman^1,2^, C. Midgley^2^, B. Sharma^1^

##### ^1^Royal Marsden NHS Foundation Trust, Sutton, UK. ^2^Saint Francis Hospice, Havering-atte-Bower, Broxhill Rd, Romford, UK

###### **Correspondence:** J. Eastman (jo.eastman@rmh.nhs.uk)

Learning Objectives

The aim of this poster is to provide an overview of common scenarios that may be encountered towards the end of life where timely imaging may be beneficial to the decision-making process.

Content Organisation

During recent years, huge advances in cancer treatment technologies have resulted in ever increasing survival statistics. However, it still remains the case that for a proportion of people who receive a cancer diagnosis, their cancer journey will end with their death. Symptoms towards the end of life may be subtle and complex and often difficult management decisions must be made by the clinical team. Even at a late stage, the use of bedside ultrasound can help inform such decisions and a best way forward for symptom control and place of care. The use of ultrasound in hospices and palliative care has increased considerably over the past decade and is now well-documented. Examples highlighting the benefits of ultrasound in these settings will be given, along with hospital based cross-sectional imaging scenarios.

Challenges can still currently be faced in the UK in obtaining imaging from out of hospital, either necessitating a decision to transfer a patient for imaging, or a decision not to image. The latter closes the door to a greater understanding of a situation, possibly to resolutions for symptom burden.

Conclusion

This overview will demonstrate how imaging can assist management decisions which can enable quality of life improvements in people facing end of life.

### P3. WB-MRI in myeloma: beyond new diagnosis

#### A. Dragan, M. Kaiser, K. Boyd, A. Riddell, D-M. Koh, C. Messiou

##### The Royal Marsden, Sutton, UK

###### **Correspondence:** A. Dragan (a.dragan@nhs.net)

Learning objectives

To review the role of whole-body MRI (WB-MRI) at diagnosis and beyond for patients with myeloma.

Content Organisation

Multiple myeloma (MM) is a clonal plasma cell proliferative disorder of the bone marrow and focal marrow lesions on MRI are now integral to diagnosis. We will present evidence and case examples for the use of WB-MRI including:

- A summary of recommended protocols (MY-RADS)
- Current imaging guidelines from the International Myeloma Working Group
- The role for WB-MRI surveillance for patients with smouldering myeloma
- WB-MRI for response assessment including detection of minimal residual disease, pseudoprogression, and heterogeneous responses in patients who have undergone multiple lines of therapy
- WB-MRI surveillance for patients with non-secretory or oligosecretory disease
- WB-MRI for confirming disease relapse

We will also discuss multicentre implementation of WB-MRI for clinical trials and advances in informatics which are likely to shape future use and application.

Conclusion

WB-MRI is established in guidelines for new diagnosis of myeloma. However, particularly as therapeutic options for patients with myeloma have advanced, there is emerging evidence and need for continued use of WB-MRI throughout the patient pathway.

### P4. Demystifying the dynamic contrast-enhanced MRI of brain

#### B. Nazir, T.S. Koh

##### National Cancer Centre, 11 Hospital Crescent, Singapore 169610

###### **Correspondence:** B. Nazir (bab_nazir@hotmail.com)

Learning objectives

To present a simplified and understandable overview of the principle, technique and interpretation of dynamic contrast-enhanced magnetic resonance imaging (DCE-MRI) and to review the most common clinical applications of DCE-MRI in the brain.

Content organisation

The capillaries and the microcirculation milieu of a normal and pathological brain have very unique and different characteristics. DCE-MRI is an important non-invasive tool that can assess these subtle haemodynamic differences. This discrimination is important in several clinical scenarios for the proper management of patients. For example, it is challenging to differentiate a “viable” brain tumour on routine MR imaging from treatment-induced changes, as both have similar appearance on conventional MR imaging. Moreover, the use of new immunotherapy anti-tumour agents can either falsely exaggerate (pseudo-progression) or diminish (pseudo-regression) the treatment response changes. We will simplify the various aspects of DCE-MRI, including:

- Pathophysiological basis of DCE-MRI; differences between the viable tumour and treatment related changes.
- Fundamentals of tracer kinetics, pharmacokinetic modelling, derived parameters and technical aspects of DCE-MRI.
- DCE-MRI characteristics of the viable tumour and the treatment-induced changes.
- Simplified approach to interpretation of DCE MRI.
- Current and emerging clinical applications.

Conclusions

DCE-MRI is emerging as an adjunct imaging tool in assessing the microvasculature signature of different brain abnormalities. A thorough understanding of this technique is essential in order to effectively use it as a problem-solving tool.

### P5. Solid pancreatic focal lesions: diagnosis and management

#### A. Veron-Sanchez^1^, M. Pezzullo^2^, I. Sameron-Beliz^3^, S. Cayon-Somacarrera^4^, N. Santamaria-Guinea^5^, M. Van Wettere^2^, M. Bali^1^

##### ^1^Institut Jules Bordet, Brussels, Belgium, ^2^Hopital Erasme, Brussels, Belgium, ^3^Hospital Universitario La Princesa, Madrid, Spain, ^4^Hospital Universitario Marques de Valdecilla, Santander, Spain, ^5^The Clatterbridge Cancer Centre NHS Foundation Trust, Liverpool, UK

###### **Correspondence:** A. Veron-Sanchez (ana.veron@bordet.be)

Learning Objectives

To provide an overview of solid pancreatic focal lesions and describe the role of imaging for disease diagnosis and patient management.

Content Organisation

Solid pancreatic focal lesion comprehends a heterogeneous wide range of benign and malignant conditions. Amongst the malignant lesions, the most common is the ductal adenocarcinoma (70-95%). Neuroendocrine tumours, acinar cell tumours, solid pseudo-papillary tumours, lymphoma, metastases and rare miscellaneous neoplasms can also be found.

Early detection and characterisation of a solid pancreatic lesion is of paramount importance in order to choose the best treatment option. However, differential diagnosis between malignant pancreatic lesions and benign inflammatory pseudo-mass such as autoimmune pancreatitis or paraduodenal pancreatitis can be very challenging.

The differential diagnosis relies very highly on the integration of clinical, laboratory and imaging findings together with pathologic information when available. Case discussion in a multidisciplinary environment represents an added value for patient management.

Dilatation of the pancreatic duct may represent one of the key findings to differentiate early stage pancreatic ductal adenocarcinoma from other solid lesions, given its ductal origin. Other imaging features to be taken into consideration are: lesion number and size, the enhancement pattern, presence of haemorrhagic/cystic components and calcifications, involvement of adjacent structures (CBD, duodenum, …) and co-existing malignant features (e.g. adenopathies, vascular involvement or distant metastases).

Conclusions

Knowledge of key imaging findings and a multidisciplinary approach are mandatory for a correct diagnosis and successful management of a solid pancreatic focal lesion.

### P6. All about peritoneal carcinomatosis: how not to miss a single case!

#### A. Veron-Sanchez^1^, C. Descatoire^1^, M. Zaiter^1^, M. Pezzullo^2^, M. Van Wettere^2^, M. Bali^1^

##### ^1^Institut Jules Bordet, Brussels, Belgium, ^2^Hopital Erasme, Brussels, Belgium

###### **Correspondence:** A. Veron-Sanchez (ana.veron@bordet.be)

Learning objectives

To depict the most frequent and the not-so-common sites and imaging features of peritoneal carcinomatosis on CT and MR.

To propose how to systematically look for peritoneal implants on CT and MR.

To describe the differential diagnosis and pitfalls.

Content organisation

The peritoneum is the second most common metastatic localisation for abdominal tumours, only surpassed by the liver. Peritoneal metastases in tumours of an extra-abdominal origin occur less frequently.

Early diagnosis of peritoneal carcinomatosis based on imaging findings, although challenging, is essential in staging and managing primary tumours. CT is usually the first diagnostic tool, but its performance is poor for subcentimetric implants and those in anatomically difficult sites. MR has proven to have a better performance due to its better contrast resolution.

The revision of 300 peritoneal carcinomatosis cases from our database (comprising abdominal and extra-abdominal tumours) has allowed us to establish a systematic diagnostic approach. Examples of the how and where to look for peritoneal implants, bearing in mind the anatomical sites and the major pathways of spread will be described, enhancing the added value of multiplanar reconstructions. Typical and less common presentations will be described using a case-based approach.

Despite the non-specificity of the imaging findings, in some cases there are helpful hints to suggest the origin of the primary tumour. Examples of pitfalls and differential diagnosis will also be discussed.

Conclusions

Early diagnosis of peritoneal carcinomatosis is paramount for patient management; therefore radiologists must be aware of its common and not-so-frequent imaging features.

### P7. Practical challenges of clinically applying mRECIST for hepatocellular carcinoma

#### A. Sachedina^1^, K. Ruchalski^2^, M. Benz^2^, R. Dewan^2^, M. Douek^2^, V. Sai^2^, J. Goldin^2^

##### ^1^Harbor UCLA Medical Centre, Carson, California, USA, ^2^David Geffen School of Medicine at UCLA, Los Angeles, CA, USA

###### **Correspondence:** A. Sachedina (asachedina@dhs.lacounty.gov)

Learning Objectives

To review the mRECIST criteria for imaging response evaluation in hepatocellular carcinoma (HCC) and discuss limitations of its clinical application.

Content Organisation

The incidence rate of liver cancer has tripled since 1980. In many countries, it is the leading cause of cancer. HCC is the most common type of liver cancer, and responsible for a significant amount of global cancer burden.

We will review the different therapies for HCC and modifications introduced to RECIST for treatment evaluation. We will discuss mRECIST criteria and potential limitations in the clinical practice including:

- Optimising image acquisition protocols
- Understanding the criteria for target and non-target lesions by mRECIST
- Potential pitfalls including:
  - ° Variable arterial contrast timing
  - ° Infiltrative HCC
  - ° Non-target malignant portal vein thrombus
  - ° New liver lesion management

Conclusion

Therapy options available for treatment of hepatocellular carcinoma is an evolving landscape. In contrast to conventional treatment, where decrease in tumour size indicates anatomic response to therapy, newer therapies often exploit the physiologic characteristics of the tumour; decrease in tumour size alone is therefore not sufficient for treatment evaluation. The introduction of the mRECIST criteria in HCC has found increasing application in clinical trials. Familiarity with image acquisition protocols, categories of treatment response, as well as imaging interpretation and potential pitfalls for categorical treatment response by mRECIST is important for practical application.

### P8. Multiparametric whole-body diffusion-weighted MRI in metastatic prostate cancer: Utility and interpretation

#### D. Lother, J. Shur, N.C. Mcaddy, D. Ap Dafydd, A. Sohaib, N. Tunariu, S.J. Withey

##### The Royal Marsden NHS Foundation Trust, Sutton, UK

###### **Correspondence:** D. Lother (dione.lother@gmail.com)

Learning Objectives

To discuss the use of multiparametric whole-body MRI (WB-MRI) in disease detection and treatment response assessment in metastatic prostate cancer. While CT and bone scintigraphy are routinely employed in prostate cancer staging, they lack sensitivity and perform poorly in the assessment of treatment response of bone marrow disease.

Content organisation

WB-MRI is superior in the detection and evaluation of metastatic bone disease and can indicate treatment response or progression before changes in macroscopic disease. WB-MRI could also have a role in evaluating tumour response in the skeleton where disease is considered unmeasurable according to RECIST and is not affected by the ‘flare’ phenomenon described on CT and bone scintigraphy. Aside from its use in the assessment of metastatic bone disease, WB-MRI is proving an increasingly important tool in the sphere of oligoprogression and oligometastasis.

We will outline:

- WB-MRI imaging protocol employed at our institution.
- Discuss the clinical interpretation of local, nodal and visceral disease.
- Review WB-MRI appearances of normal bone marrow, active bone marrow disease and treatment response patterns of bone marrow disease in metastatic prostate cancer.
- Outline limitation and pitfalls of WB-MRI.

Conclusion

WB-MRI is an emerging imaging tool that has proven superiority in the detection and assessment of metastatic prostate cancer involving the bone marrow. WB-MRI can indicate early treatment response or progression and is proving an important tool in the setting of oligometastasis and oligoprogression, allowing targeted therapies to sites of active disease.

### P9. Gallbladder neoplasms: Imaging spectrum and role of the radiologist in diagnosis and management

#### A. Chandra, S. Mukhopadhyay, A. Chatterjee, P. Ghosh, A. Gehani, S. Sen

##### Tata Medical Center, Kolkata, India

###### **Correspondence:** A. Chandra (draditichandra@gmail.com)

Learning objectives

To review the spectrum of imaging findings of gallbladder carcinoma and atypical pre-malignant neoplasms.

To review staging in gallbladder carcinoma.

To review the role of the radiologist in management of gallbladder cancer including the role of interventions as a palliative, as well as bridging, therapy to surgery.

Content organisation

Gall bladder carcinoma is a rare aggressive malignancy. It is prevalent in certain geographic regions and ethnic groups. Optimum imaging is key to early diagnosis and management. We will discuss imaging findings of gallbladder carcinoma, as well as pre-malignant conditions. Various morphological types of gallbladder carcinoma will be depicted along with differentials. Staging of gallbladder cancer as well as controversies in nodal staging will be discussed. Imaging strategies for incidentally diagnosed gallbladder carcinoma will be presented. Interventions as a palliative procedure and bridge to definitive therapy for e.g. biliary drainage and portal vein embolisation will be emphasised.

Conclusions

Role of the radiologist in gallbladder neoplasms is multifold and key to management of these conditions. Gallbladder carcinoma management defined the role of the radiologist as an imaging physician.

### P10. Osteosarcopenia: A new geriatric syndrome with great impact on cancer patients

#### J. Huh

##### University of Ajou College of Medicine, Suwon, Gyeonggi Province, South Korea

###### **Correspondence:** J. Huh (jimihuh.rad@gmail.com)

Learning objectives

1. To explain the concept and updated knowledge about osteosarcopenia.
2. To systematically review the current research about impact of osteosarcopenia on cancer patients.

Content organisation

We will review the spectrum of sarcopenia derivatives including:

- Concept and updated knowledge about osteosarcopenia which is a combination of sarcopenia and osteoporosis.
- Changing concepts of sarcopenia derivatives: sarcopenia, myosteatosis, and osteosarcopenia.
- Distinct pathophysiology of osteosarcopenia focusing on interaction between muscle and bone.

We will systematically review the clinical impact of osteosarcopenia on various cancer patients:

- Prognostic parameter for mortality and overall survival in cancer patients.
- Independent risk factors for osteoporotic fractures and frailty in cancer patients.

We will discuss body composition imaging for evaluation of osteosarcopenia:

- Current leading diagnostic modalities of osteosarcopenia : CT, MRI and DXA
- Imaging methods to measure the skeletal muscle area (SMA) and bone mineral density (BMD)
- Evolution of AI techniques: from 2D segmentation to AI segmentation and pattern analysis for BMD

Conclusions

Osteoporosis, a combination of osteoporosis and sarcopenia, is a distinct disease entity with unique pathophysiology. Osteosarcopenia is highly associated with frailty and mortality in cancer patients. Advances in imaging have enabled detailed analysis of osteosarcopenia.

### P11. Prostate cancer staging using molecular imaging

#### L. Chinthakunta^1^, T.S.C. Ng ^2^, H. Shah^3^

##### ^1^Brigham and Women's Hospital, Dana-Farber Cancer Institute, Boston, MA, USA, ^2^Massachusetts General Hospital, Boston, MA, USA, ^3^Brigham and Women's Hospital, Dana-Farber Cancer Institute, Boston, MA, USA

###### **Correspondence:** L. Chinthakunta (doclokesh0908@gmail.com)

Learning objectives

To review the role of molecular imaging in staging prostate cancer.

Content organisation

Prostate cancer is one of the most common cancers among men all over the world. Accurate staging of these patients is important for appropriate prognostication and treatment strategy choice. Imaging plays a key role in this pathway. In particular, molecular imaging, especially with the advent of novel PSMA-targeted PET tracers, is poised to play an ever more important role. Here, we will review the role of imaging for PCa staging, with an emphasis on novel PET tracers for this purpose.

The outline of the exhibit is as follows:

- Staging of prostate cancer - NCCN guidelines.
- Current imaging for staging.
  - ° CT, MRI
  - ° Molecular imaging
    - ■ Bone scintigraphy
    - ■ Fluciclovine
  - ° Novel PSMA-targeted agents
- Utility of molecular imaging for staging in different disease settings.

Conclusions

Molecular imaging is an integral tool in the management of prostate cancer. It provides important information on staging at the time of initial diagnosis, in the setting of biochemical recurrence, and selecting an appropriate treatment strategy. With radiolabelled PSMA, there has been an expanded role of nuclear medicine, not only in diagnosis but also in therapeutics.

Prostate cancer staging using molecular imaging

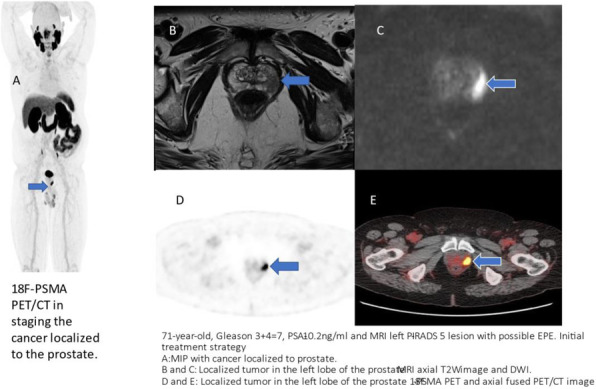

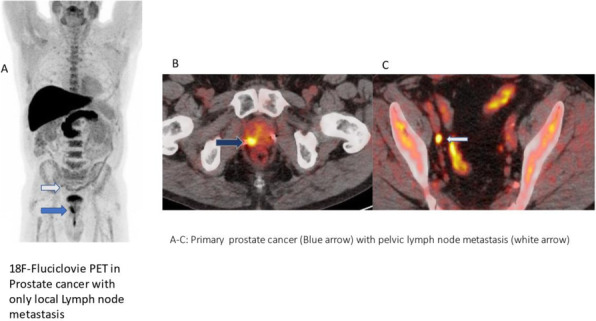

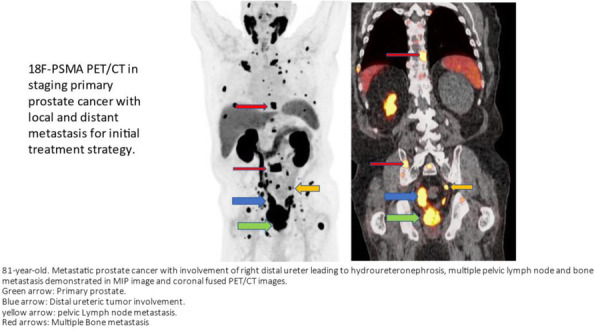

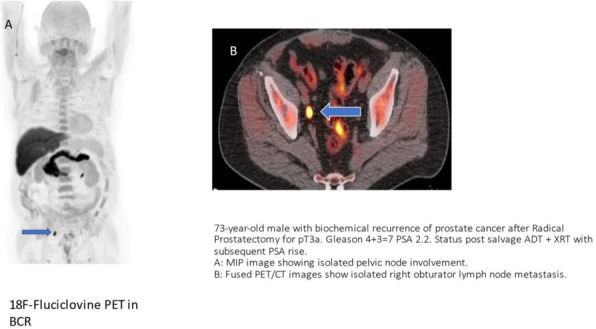

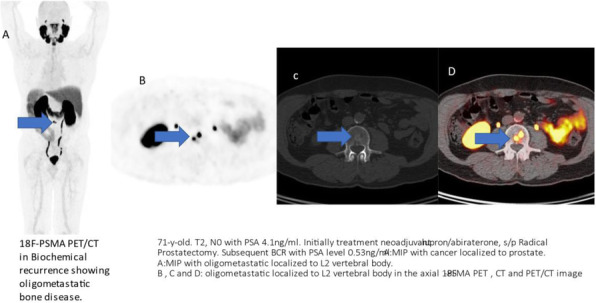

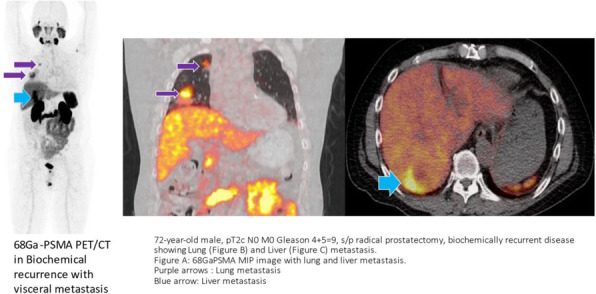

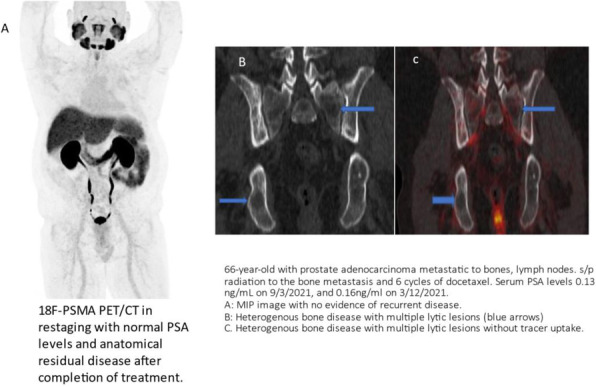

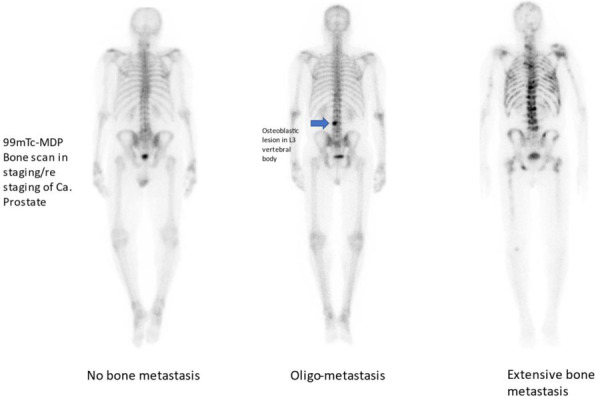

















### P12. Utilisation of Artificial Intelligence (AI) aided body composition imaging for cancer patients

#### K.W Kim^1^, Y. Ko^1^, Y. Shin^1^, J. Lee^2^, J. Lee^3^, J. Huh^4^

##### ^1^Asan Medical Center, Korea, ^2^School of Computer Science &amp; Engineering, Soongsil University, Korea, ^3^Biomedical Engineering, Kyung Hee University, Korea, ^4^Ajou University Hospital, Korea

###### **Correspondence:** K.W Kim (medimash@gmail.com)

Learning objectives

Body composition imaging is important to improve holistic patient care and to achieve survival benefit in various cancer patients. Artificial Intelligence (AI) enables us to perform automatic body composition measurements in opportunistic CT scans and to detect patients with sarcopenia and osteoporosis.

Content organisation

We will review the evolution of AI techniques for fully automatic body composition measurements on opportunistic CT scan, as follows:

- Automatic 2D and 3D segmentation for skeletal muscle area, visceral fat area, and bone mineral density.
- Automatic diagnosis/classification for sarcopenia, sarcopenic obesity, myosteatosis, and osteoporosis.
- Automatic L3 lumbar vertebral level selection.

We will present the work process to utilised AI-aided body composition imaging for clinical research, as follows:

- Work process from research design, AI-aided body composition measurement, data analysis, and writing papers.
- Introduction of 36 papers which have used AI-aided body composition imaging in cancer patients.
- Utilisation of body composition imaging in clinical trials for new drug development.

Conclusions

AI-aided measurement of skeletal muscle area, visceral fat area, and bone mineral density simultaneously on opportunistic CT scans is greatly helpful to evaluate sarcopenia, obesity, myosteatosis, osteoporosis and to improve cancer patient care.

### P13. Sarcoid-like reaction among challenging mimickers

#### A. Kovic, C. Frías, C .Carrera, S. De Luca

##### Hospital Alemán, Buenos Aires, Argentina

###### **Correspondence:** S. De Luca (sdeluca@hospitalaleman.com)

Learning objectives

Point out the importance of this entity when interpreting its imaging findings in a cancer patient, without symptoms or signs suggestive of systemic sarcoidosis.

Content organisation

Sarcoidosis is a systemic granulomatous disorder of unknown origin, which is characterised by chronic non-caseating granulomatous inflammation. These granulomas can mimic tumour progression in oncologic patients, without symptoms or signs suggestive of systemic sarcoidosis.

This entity has been described not only with lymphoma but with various solid organ malignancies including lung cancer, breast cancer, colorectal, and genitourinary cancer The most common pattern is mediastinal nodal and/or symmetric hilar uptake. Another presentation includes pulmonary abnormalities such as pulmonary nodules, ground glass opacities, regional lymph nodes, liver, spleen or bone marrow uptake.

Conclusions

In patients with complete remission of malignancies, newly developed bilateral hilar and mediastinal lymphadenopathies with or without pulmonary nodules of perilymphatic distribution, in the absence of recurrence at the primary tumour site and extrathoracic sites, may suggest a sarcoid-like reaction. Such cases warrant histologic evaluation of the lymph nodes to prevent unnecessary systemic chemotherapy.

### P14. 18-F FDG PET/CT in the evaluation of neurolymphomatosis

#### E. Franquet^1,2^, L Chinthakunta^1,2^, C. Sakellis^1,2^, H. Shah^1,2^

##### ^1^Department of Imaging, Dana Farber Cancer Institute, Boston, MA, USA, ^2^Department of Nuclear Medicine and Molecular Imaging, Brigham and Women's Hospital, Boston, MA, USA

###### **Correspondence:** E. Franquet (e.franquetelia@gmail.com, elia_franquet@dfci.harvard.edu)

Learning objectives

- Discussion of neurolymphomatosis (NL) and its clinical presentation.
- Review imaging characteristics of neurolymphomatosis on FDG PET/CT and other modalities with illustrative cases.
- Review diagnosis and treatment options for NL.

Content Organisation

Neurolymphomatosis (NL) represents infiltration of the central nervous system or the peripheral nervous system by malignant lymphoma cells and is an uncommon presentation of non-Hodgkin lymphoma (NHL). Nerve roots, brachial and lumbosacral plexuses, and peripheral nerves may be involved. Prompt identification of NL is crucial for timely initiation of treatment.

We will discuss:

- Frequency of presentation and most common lymphoma types associated to NL.
- Clinical presentation.
- Role of fluorodeoxyglucose (FDG) positron emission tomography/computed tomography (PET/CT) to evaluate NL (presence and extent of neuronal involvement, identify potential targets for biopsy, and response to therapy).
- Imaging characteristics of NL on FDG PET/CT compared to other neurological entities with representative examples of different management, including diagnostic gold standard and treatment.

Conclusion

Neurolymphomatosis (NL) represents infiltration of the central nervous system or the peripheral nervous system by malignant lymphoma cells and is an uncommon presentation of non-Hodgkin lymphoma (NHL). FDG PET/CT plays an important role in assessing disease status and can detect neural involvement if present. High degree of suspicion, early detection and early treatment are crucial in NL to help guide therapy.

### P15. MRI and multidisciplinary team discussion in pre-treatment rectal cancer assessment: Kenyan transitional experience

#### T.M. Mutala, F.A. Anyumba, A. Odhiambo, J.M. Githae, W. Waweru

##### University of Nairobi, Nairobi, Kenya

###### **Correspondence:** T.M. Mutala (musilamutala@gmail.com)

Aim

To establish the diagnostic accuracy of MRI in assessment and staging of rectal cancer (RC) as well as compliance to multidisciplinary team (MDT) discussion before treatment during transition to national guidelines that recommended the use of both approaches.

Methods

A cross-sectional study with a sample size of 48 participants identified during clinical workflow with endoscopically biopsied RC was carried out between October 1, 2020, and January 31, 2021. MRI was the index test while post-surgical histopathology was the reference standard. The number of patients presented in multidisciplinary team (MDT) meetings was also determined.

Results

The mean age of the patients was 52.3 (SD 14.1) years. Overall diagnostic accuracy was 91.67% for differentiating T1/T2 from higher stages, 88.46% for circumferential margin (CRM) involvement and 70.8% for nodal disease. Only 8 out of 48 (16.6%) of patients were discussed at an MDT before their surgical management. A significant number of patients (81.25%) who had MRI T3/T4 disease did not benefit from the imaging findings since they still underwent upfront surgery.

Conclusion

The overall diagnostic accuracy of MRI in RC was comparable to globally established figures in differentiating between early (T1/T2) disease from more locally advanced rectal cancer, CRM determination and nodal disease. Uptake of pre-surgery MDT discussion was low which can stifle the benefits of pre-treatment MRI assessment.

Consent to publish

Institutional Ethics Committee approval and participants’ consent were obtained.

### P16. Retrospective assessment of breast tumours in neo-adjuvant setting: Radiomics approach

#### A. Acquisto, M. Radermacker, S. Picchia, S. Chkili, M. Zaitar, M. Pezzulo, M-A. Bali

##### Institut Jules Bordet, Brussels, Belgium

###### **Correspondence:** A. Acquisto (anais.acquisto@bordet.be)

Aim

Breast cancer has the highest incidence among cancers in women worldwide. Neoadjuvant therapy has been established especially for locally advanced breast cancer to downstage the disease. Radiomics is a new field of research, based on the concept that conventional biomedical images contain information that may reflect underlying pathophysiology and their relationship could be revealed via quantitative image analysis. We aimed to assess radiomics approaches predictive of complete pathological response and breast cancer subtypes.

Materials and Methods

We retrospectively assessed 39 patients with breast tumours treated with neoadjuvant chemotherapy and surgery. IVIM and DCE MR maps were analysed. Lesion segmentation was performed semi-automatically to obtain volume of interest (VOI). Syngo.via Frontier CT Radiomics (Siemens Healthcare) was used to obtain radiomics features according to statistical and machine learning approaches.

Results

Radiomics features were predictive for complete pathological response and cancer subtypes. The statistical approach (multivariate analysis) performed better than machine learning to predict compete response: AUC 0.86 for multivariate analyses vs 0.77 for machine learning; and cancer subtypes (luminal subtype: AUC 0.94 for multivariate analyses vs 0.85 for machine learning; HER2 receptor subtype: AUC 0.61 for multivariate analyses and AUC 0.47 for machine learning; triple negative AUC 0.86 for multivariate analyses and AUC 0.79 for machine learning).

Conclusions

Radiomics features obtained using statistical and machine learning approaches showed to be predictive of pathologic complete response and breast cancer subtypes in patients treated in a neo-adjuvant setting and four radiomics signatures were identified.

### P17. Diagnostic efficiency of elastography-determined apparent margin thickness of breast tumours in predicting histological grade

#### A. Paluri, S.Sampangi, S.S. Shivalingappa

##### HCG Hospitals, Bangalore, India

###### **Correspondence:** A. Paluri (anushap10@gmail.com)

Background and Aim

One of the most important factors in determining the overall prognosis of breast malignancies is the histological grade of the tumour. This study aims to assess the association between ultrasound elastography determined apparent margin thickness of breast tumours, their histological grade and Ki-67% expression.

Methods

This retrospective study was performed at a tertiary healthcare facility in Bengaluru which included 50 patients with proven breast malignancy, who were initially evaluated by ultrasonography, elastography and later underwent breast conservative surgery or mastectomy with axillary dissection. Patients with prior breast surgery were excluded from the study. The elastography-determined apparent margin thickness of tumour was calculated by subtracting the dimension of tumour on B-mode image from elastogram. Apparent margin thickness was then correlated with the histological grade (using Nottingham histological score), Ki-67% expression and other pathological features like presence of lymphovascular invasion and axillary nodal metastases.

Results

Grade III tumours and tumours with high Ki-67% showed statistically significant association with higher apparent tumour margin thickness determined by elastography compared to the lower grade tumours (p value <0.05). There was also significant correlation between the apparent tumour margin thickness, lymphovasular invasion and axillary nodal metastases.

Conclusion

The ultrasound elastography-determined apparent tumour margin thickness aids in predicting histologically higher-grade breast tumours with high Ki-67% expression and thereby has the potential to play a key role in staging and patient management.

### P18. Value of systematic prostate biopsy for detecting prostate cancer in biopsy-naïve patients with negative mp-MRI

#### V. Kumar, J. Yu, S. Winks

##### Virginia Commonwealth University Health System, Richmond, VA, USA

###### **Correspondence:** V. Kumar (kumarvk@vcu.edu)

Aim

Necessity of systematic transrectal ultrasound (TRUS)-guided prostate biopsy for detection of clinically significant prostate cancer (csPCa) after negative multiparametric-MRI of the prostate (mp-MRI) has been highly debated. This study aims to evaluate the potential value of TRUS-guided prostate biopsy for detecting csPCa in biopsy-naïve patients with elevated PSA and negative mp-MRI.

Methods

A single-institution, retrospective study was performed. Records of 177 biopsy-naïve patients with elevated PSA and negative mp-MRIs who underwent subsequent systematic prostate biopsies over an 8-year period (2014-2022) were reviewed. Clinical and histopathological information, such as age, PSA, PSA density, prostate volume, csPCa presence, Gleason score (GS), and percent positive cores (PPC) was recorded. Two genito-urinary radiologists were blinded to the biopsy results and retrospectively reviewed the mp-MRI studies for cancer suspicious regions (CSR) using PI-RADS v2.

Results

Of 177 patients, 12% (n=22) had csPCa (GS >7) proven by TRUS-guided biopsy. Patients with csPCa had the following mean values: age (60.5 years), PSA (6.65 ng/mL), prostate volume (37.2 cc), and PSA density (0.21 ng/mL^2^). Patients with csPCa were divided into groups based on GS: GS 7 (n=18), GS 8 (n=3), and GS 9 (n=1). Mean PPCs were 34%, 28% and 50% respectively. Retrospective review yielded 3 CSRs (each PI-RADS 3) from the csPCa cases (14%).

Conclusions

Systematic TRUS-guided prostate biopsies are diagnostically useful even after a negative mp-MRI in biopsy-naïve patients with elevated PSA. In this study, mp-MRI missed 12% of csPCa in this population, with no or only mildly suspicious mp-MRI findings on retrospective review.

### P19. FDG PET/CT semi-quantitative parameter (SUVmax) may not predict treatment response in non-Hodgkin lymphoma patients correctly

#### A. Doroudinia, M. Bakhshayesh Karam, B. Samimi

##### Chronic Respiratory Diseases Research Centre, National Research Institute of Tuberculosis and Lung Diseases (NRITLD), Shahid Beheshti University of Medical Sciences, Tehran, Iran

###### **Correspondence:** A. Doroudinia (abtin1354@gmail.com)

Purpose/Background

Non-Hodgkin lymphoma (NHL) is amongst those tumours in which FDG PET/CT scan has established its role in disease aspects including initial staging, treatment response evaluation and restaging. Recently semi-quantitative parameters other than SUVmax are gaining attention in treatment response evaluation. This current study aims to evaluate the value of SUVmax parameter in treatment response evaluation in NHL patients.

Methods

In this retrospective cross-sectional study, we evaluated 900 NHL patients including 540 cases of diffuse large B-cell lymphoma (DLBCL). All patients were followed for at least one year and had FDG PET/CT scan for initial staging and also treatment response evaluation after completion of standard chemotherapy. Patients who received radiotherapy were excluded from the study. Complete metabolic response was attributed to patients with Deauville score 1- 3.

Results

The most common site of tumoural involvement was the mediastinum and chest (25%) and 40% had mixed organ tumoural involvement.

On subsequent evaluation, the rate of disease progression was 10% (most common in chest), stable disease was determined in 17% (most common in head and neck), partial response (PR) recognised in 33% (most common in chest) and complete metabolic response (CR) was seen in 40% (most common in abdomen and pelvis).

Based on receiver operating characteristic curve (ROC) analysis, the value of SUVmax itself and its changes before and after treatment was not able to differentiate between CR and PR cases (p=0.15).

Conclusion

Value of SUVmax had insufficient predictive value for successful assessment of response to treatment in patients with NHL.

Financial disclosure

Authors of this manuscript acknowledge that they have all contributed to this work significantly.

Authors of this manuscript acknowledge that they all agree with the content of this work.

Authors of this manuscript declare that there is no funding or conflict of interest for this work.

### P20. To demonstrate the various pitfalls of PET in onco-imaging from head to toe

#### N. Alemao, S. Swamy, A. Kesari, M. Kumar, S. Sampangi, K. Kallur

##### HCG Hospital, Bangalore, India

###### **Correspondence:** N. Alemao (niyarahalemao99@ymail.com)

Aim

To demonstrate the various pitfalls of PET in onco-imaging using a pictorial representation of PET images captured from head to toe.

Method

Positron emission tomography scans of around 90 patients visiting our hospital were selected. These included ^68^Gallium-FAPI, 18-F FDG, ^68^Gallium-PSMA, ^68^Gallium-dotanoc and DOPA PET scans. A note was made of artefacts, physiological uptake, benign conditions and tumour mimics generating false-positive PET/CT results. Malignancies included brain glial tumours, head and neck squamous cell and adenocarcinomas, lymphoma, melanoma, multiple myeloma, lung carcinomas, sarcomas, gastrointestinal, genitourinary and ovarian malignancies, carcinoma endometrium and cervix, osseous neoplasms, neuroendocrine tumours, adrenal neoplasms and carcinoma of the prostate.

Conclusion

The most common pitfalls of PET imaging include blood glucose levels or psychotropic drugs that greatly affect the physiological FDG uptake. False-positive PET/CT interpretation pitfalls were mostly due to high FDG uptake by physiological causes, benign thyroid nodules, unilateral cranial nerve palsy and increased FDG uptake due to inflammation, recent chemoradiotherapy and surgery. False-negative findings are caused by lesion vicinity to structures with high glucose metabolism, obscuration of FDG uptake by dental hardware, inadequate PET scanner resolution and inherent low FDG-avidity of some tumours. Normal distribution of FDG uptake in children differs from adults. Uptake of FDG may be seen in numerous benign conditions, including inflammation, infection and trauma. Although PET imaging is a force to reckon with, an understanding of the pitfalls is essential for clinical judgement of using suitable alternatives and appropriate selection of pharmaceuticals is required to deliver optimum patient care.

### P21. ^68^Gallium-FAPI versus 18-F FDG PET scan in imaging of gynaecological malignancies

#### N. Alemao, S. Swamy, A. Kesari, M. Kumar, S. Sampangi, K. Kallur

##### HCG Hospital, Bangalore, India

###### **Correspondence:** N. Alemao (niyarahalemao99@ymail.com)

Aim

To assess the diagnostic value of ^68^Ga-FAPI and 18-F FDG PET scan in the diagnosis and staging of primary malignancies.

Methods

PET has shown to be useful in endometrial carcinomas to stratify risk with a positive correlation between SUV_max_ and histological grade. Staging of the disease is necessary for accurate management of the patient. While PET has been studied in combination with CT and MRI in evaluation of these lesions using FDG, the use of FAPI has not been popular.

This study included a total of 35 patients. Inclusion criteria was determined by post-surgical histology or biopsy characterisation of the lesion. Malignancies included malignant ovarian epithelial neoplasms, carcinoma of the endometrium, uterine sarcomas, malignant mixed Mullerian tumours, adenocarcinoma of the cervix, squamous cell carcinoma of the cervix and carcinoma of the vagina. Exclusion criteria included benign tumour mimics and neoplasms and histopathological unproven cases.

Results

In comparison with previous studies using FDG (18-fluorodeoxyglucose) as the radiotracer, 18F FDG performs better than ^68^Ga-FAPI in detection, staging and restaging of primary tumour in carcinoma of the endometrium, uterine sarcomas, malignant mixed Mullerian tumours, adenocarcinoma and squamous cell carcinoma of the cervix. In cases of ovarian adenocarcinomas with peritoneal carcinomatosis, FDG PET tends to diagnose the peritoneal deposits, while the primary tumour remains veiled. Secondary to peritoneal invasion of the tumour, a fibrotic response may occur which showed increased uptake of the radiotracer ^68^Ga-FAPI.

Conclusion

FDG PET scans have a higher sensitivity in uterine and cervix carcinomas.

^68^Ga-FAPI is superior than 18-F FDG PET in imaging of primary ovarian neoplasms

### P22. Prognostic significance of FDG-PET in oesophageal cancer subtypes – an unseasoned tool

#### N. Alemao, S. Swamy, A. Kesari, M. Kumar, S. Sampangi, K. Kallur

##### HCG Hospital, Bangalore, India

###### **Correspondence:** N. Alemao (niyarahalemao99@ymail.com)

Aim

1. To identify the SUV max associated with oesophageal cancer subtypes.
2. To demonstrate the prognostic significance of FDG-PET in oesophageal cancer subtypes.

Methods

Oesophageal cancer is not the most common of the various neoplasms seen. Statistically, it forms about 1% of all cancers that affect humans and less than 10% of the cancers that involve the entire gastrointestinal tract. But it is known for its very poor survival rate and high morbidity.

The study was conducted in patients with oesophageal cancer who had a PET study and further underwent a histopathological analysis to determine the subtype. The SUV max of each patient was obtained and a comparison was done with the histopathological subtype to determine the prognosis of the patient. Furthermore, lymph nodal and distant metastasis for each histological subtype and location of primary tumour was noted.

Results

Mean tumour SUVmax was less for the adenocarcinoma group in comparison to squamous cell carcinoma group (p<0.005). Also, higher SUV values represented poorer prognosis.

The higher the SUV values, the higher was the tumour size and T stage of the primary tumour. Possibility of lymph nodal and solid organ metastasis was also higher with higher SUV max values of the primary tumour.

Conclusion

In the present study, it was found that the prognosis of patients with high SUVmax at baseline is significantly worse than that of patients with low SUVmax. Prognosis of the patient was better with lower SUVmax values.

Ethics Committee approval

Obtained approval from the Institutional Ethics Committee

### P23. Intraparenchymal blood patch technique after CT-guided biopsy of intrathoracic lesions minimises risk of pneumothorax

#### Binoy Kumar Choudhury

##### Dr B Borooah Cancer Institute

###### **Correspondence:** Binoy Kumar Choudhury (choudhury60@gmail.com)

Aim

To determine whether intraparenchymal blood patch technique after CT-guided biopsy of deep seated lung and mediastinal lesions can minimise and prevent the risk of pneumothorax.

Methods

42 lung and mediastinal lesions (between August 2018 to July 2021) having biopsy path of at least 2 cm aerated lung were selected so that an effective blood patch seal might be applied. Biopsy was performed under CT guidance using a coaxial system. A 19G guiding needle was used and a 20G spring-loaded semi-automatic core biopsy gun was used to obtain samples.

Biopsy gun was removed and depending on the length of biopsy tract from the tip of the needle to pleura, between 3-8 ml of the patient’s blood (non-clotted or partially clotted) was injected into the needle tract while the guiding needle was withdrawn, filling the entire needle tract from the biopsy site to the visceral pleura. CT scan was performed in that position to see evidence of pneumothorax. Chest skiagram was performed two hours after the procedure to see any pneumothorax.

Results

Intraparenchymal blood patch technique after biopsy was performed in 42 deep-seated and risky intrathoracic masses ranging from 10 mm to 27 mm in size in the age group of 33-70 years. Two patients developed very small pneumothoraces. Post-procedure CT revealed very small pneumothoraces and these were not seen in chest skiagrams taken two hours after the procedure.

Conclusion

Use of intraparenchymal blood patch after biopsy of deep-seated lung and mediastinal lesion significantly reduces and prevents the risk of pneumothorax.

### P24. Image-guided percutaneous biopsy of pancreatic masses: 5-years’ experience in a tertiary cancer centre

#### Binoy Kumar Choudhury

##### Dr B Borooah Cancer Institute, Guwahati, India

###### **Correspondence:** Binoy Kumar Choudhury (choudhury60@gmail.com)

Aim

The purpose of this study was to evaluate the safety and diagnostic accuracy of CT and ultrasound (US)-guided biopsy in pancreatic masses.

Method

It was a retrospective study of patients referred for image-guided biopsy of pancreatic masses from January 2017 to December 2021. CT or US-guided core biopsy or FNAC or both were performed, based on CT and US findings. FNAC was performed using a spinal or Chiba needle. Core biopsy was performed using a coaxial system. A 17 or 19 gauge guiding needle was used, and 18 or 20 gauge spring-loaded semi-automatic core biopsy gun was used.

Results

A total of 55 patients underwent image-guided pancreatic biopsy using CT or US as image guidance. US guidance was used in 20 cases and CT guidance was used in 35 cases. The mean pancreatic size was 3.1 cm. The majority of the lesions were located in head (58.2%), followed by body (23.6%) and tail (18.2%). Direct access was performed in 32 cases (58.2%) and in the other 23 cases (41.8%), indirect approach (mostly transgastric or transhepatic) was used. FNAC only was performed in 15 cases (27.3%), and both core biopsy with FNAC were obtained in 40 cases (72.7%). A conclusive result was obtained in 49 cases (89%). There were no major complications in our study.

Conclusion

Image-guided percutaneous needle biopsies are a safe and highly effective diagnostic technique with excellent accuracy for patients with pancreatic masses.

